# Lys-specific gingipain (Kgp) of *P. gingivalis* promotes viral infection by disabling the interferon pathway

**DOI:** 10.1128/mbio.00298-25

**Published:** 2025-08-28

**Authors:** Ewelina Dobosz, Anna Golda, Michal Kanoza, Weronika Kowalczuk, Barbara Potempa, Jan Potempa, Anna Gasiorek, Natalia Madeja, Joanna Budziaszek, Danuta Mizgalska, Tulay Yucel-Lindberg, Joanna Koziel

**Affiliations:** 1 Department of Microbiology, Faculty of Biochemistry, Biophysics and Biotechnology, Jagiellonian University 98817, Krakow, Poland; 2 Department of Dental Medicine, Division of Pediatric Dentistry, Karolinska Institutet 27106, Huddinge, Sweden; 3 Doctoral School of Exact and Natural Sciences, Jagiellonian University, Kraków, Poland; 4 Department of Oral Immunity and Infectious Diseases, University of Louisville School of Dentistry, University of Louisville 162193, Louisville, Kentucky, USA; University of North Carolina at Chapel Hill, Chapel Hill, North Carolina, USA; University of Pittsburgh School of Medcine, Pittsburgh, Pennsylvania, USA

**Keywords:** periodontitis, *Porphyromonas gingivalis*, herpes simplex virus, latency, interferon signaling

## Abstract

Periodontitis (PD) is a chronic inflammatory disease of the periodontium with a high prevalence and is considered a potential risk factor for the development of other diseases. These include viral infections of the upper and lower respiratory tracts, including those caused by the *Herpesviridae* family, human immunodeficiency virus, hepatitis C and B viruses, influenza virus, and severe acute respiratory syndrome coronavirus 2 (SARS-CoV-2), which have been identified with greater frequency in patients with PD. The underlying molecular mechanisms that underpin this comorbidity remain to be elucidated; however, the compromised capacity of the oral mucosa-associated antiviral response is a plausible explanation. Driven by clinical data that revealed the *Herpesviridae* family as the most commonly identified viruses in PD patients, this study was aimed to determine the effect of *Porphyromonas gingivalis*, the key etiological factor in periodontitis, on the development of herpes simplex virus-1 (HSV-1) infection. Using a model of the human gingiva, it was demonstrated that *P. gingivalis* significantly increases infection with HSV-1, promoting tissue distribution and propagation of the virus. This phenomenon can be attributed to the impairment of the interferon response, a consequence of proteolytic modifications of major signaling components catalyzed by Kgp gingipain. Furthermore, *P. gingivalis* infection has been observed to promote reactivation of HSV-1 in neuronal cells but via IFN-independent mechanism. These findings, demonstrating the attenuation of the host defense, expand our basic knowledge of the mechanisms underlying polymicrobial infections and clarify the observed comorbidity of PD with viral disorders.

**IMPORTANCE:**

Periodontitis (PD) is a chronic inflammatory disease of the gingiva, with a high prevalence. Clinical reports indicate the significant role of PD in the development of comorbidities, including *Herpesviridae* infections; however, the molecular basis of this phenomenon has not yet been described. In our work, we uncovered a novel molecular mechanism by which the interferon-dependent antiviral response is tailored by the cysteine protease of *P. gingivalis*—Kgp. Using gingival keratinocytes and a model of human gingiva, we have demonstrated that lysin-specific gingipain attenuates the antiviral response and promotes the propagation of herpes simplex virus-1, which is one of the most frequently identified viruses in patients suffering from PD. These findings expand our knowledge of the mechanisms underlying polymicrobial infections and may provide a basis for considering PD as a gateway to viral infection.

## INTRODUCTION

Periodontitis (PD) is an inflammatory disease of bacterial etiology, leading to the destruction of teeth-supporting tissues and, eventually, bone loss (1). The initiation of this disease is marked by the formation of a subgingival biofilm, which establishes a unique niche for the growth of pathogenic anaerobic species (2). Recent clinical data demonstrate a strong association between periodontitis and an elevated risk of developing viral infections (3, 4). Higher prevalence and more frequent reactivation of latent viral infections are observed in PD patients (4, 5). Infections caused by human immunodeficiency virus (HIV), hepatitis C and B viruses (HCV, HBV), influenza virus (IVA/B), and severe acute respiratory syndrome coronavirus 2 (SARS-CoV-2) tend to be more severe in patients suffering from periodontal diseases (6–9). However, the results of randomized multicenter studies indicate that herpesviruses are the most prevalent viruses co-occurring in patients with periodontitis (3, 10–12). Although clinical data clearly indicate the comorbidity between these diseases, the molecular basis of this relationship remains poorly understood.

*Porphyromonas gingivalis* has been identified in the multispecies, dysbiotic biofilm formed in the subgingival surface of teeth of patients with periodontal disease (1). This keystone pathogen compromises the homeostasis of the mucosal immune system, resulting in uncontrolled inflammation (13). Of particular importance in this process are cysteine proteases, also known as gingipains. These enzymes have the capacity to rapidly cleave both extracellular and intracellular proteins, including those that determine appropriate activation of cell signaling pathways and the efficient response to infection (14, 15). The impact of gingipains on cell signaling is well documented, with research highlighting their ability to cleave extracellular receptors, including those which recognize endotoxins (15–17). However, there is a paucity of research about the influence of gingipains on intracellular sensors of pathogen-associated molecular patterns and executive molecules activated by these receptors, including those dedicated for nucleic acids and other viral components (18, 19). The recognition of viral particles determines targeted antiviral, interferon-mediated responses, and the influence of periodontopathogens on this process is barely investigated. Some data reported that infection with *P. gingivalis* leads to suppressed expression of several transcription factors initiating interferon production, including interferon regulatory factors, IRF1, IRF3, IRF7, and IRF9, and to proteolytical degradation of IFN receptors (20). Moreover, it has been demonstrated that exposition to periopathogens can impede the nuclear translocation of the interferon-stimulated gene factor-3 (ISGF-3) complex, consequently diminishing the antiviral response (20). Conversely, *P. gingivalis* has been observed to induce uncontrolled interferon production through the degradation of MyD88 and the activation of the cGAS-STING signaling pathway (18, 21). Consequently, there is an evident necessity for a more in-depth, molecular-based investigation into the mechanisms by which periopathogens regulate the interferon response.

The objective of the present study was to ascertain the role of *P. gingivalis* in interferon-dependent antiviral response. The results obtained from this research demonstrated that *P. gingivalis* infection led to the abrogation of the interferon signaling pathway through a Kgp-dependent proteolysis. As a consequence of impairment of mucosa defense, an enhanced herpes simplex virus-1 (HSV-1) infection of oral mucosa was observed. Furthermore, it was found that this directly led to the reactivation of latent viruses in neuronal cells. The findings of this study elucidate a novel molecular pathway through which *P. gingivalis*-induced impairment of the antiviral response may occur, thereby explaining the heightened susceptibility to viral infections observed in patients suffering from periodontitis.

### Key findings

*P. gingivalis* impairs antiviral, interferon-dependent response via targeted intracellular proteolysis.

The process is catalyzed by the lysine-specific protease, gingipain Kgp.

Attenuation of antiviral response led to increased multiplication of HSV-1 in oral epithelial cells.

Kgp leads to IFN-independent activation of the latent form of the virus in neurons.

Kgp inhibition should be considered as preventive strategy against viral infections.

Periodontitis is a risk factor of viral infection development.

## Results

### *P. gingivalis* infection affects interferon response of gingival epithelium

**Fig 1:**
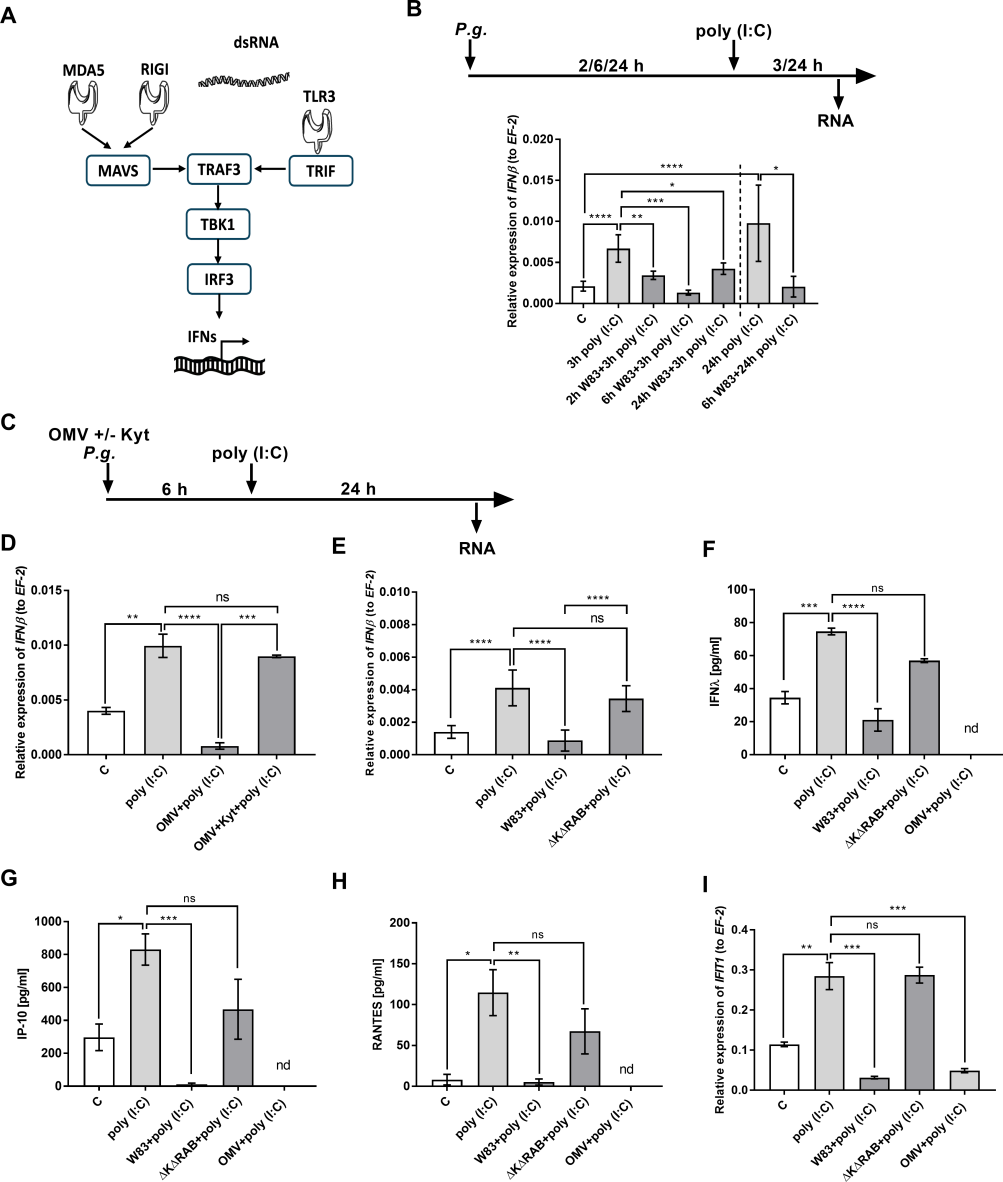
*P. gingivalis* infection affects the interferon response of gingiva epithelium. (**A**) The scheme depicts the initiation of IFN production upon nucleic acid recognition. Prepared using Servier Medical Art. (**B**) Gingival keratinocytes were exposed to *P. gingivalis* W83 strain for 2, 6, or 24 h and then incubated with 10 µg/mL poly (I:C) for additional 3 or 24 h. *IFN*β expression was estimated using quantitative real-time PCR (qPCR) and compared to control [C], not stimulated cells. (**C**) Telomerase-immortalized gingival keratinocytes were exposed to (**D**) OMV and/or OMV previously incubated with KYT inhibitors or infected with (**E**) *P. gingivalis* W83 strain and ∆K∆RAB strain, and after 6 h, 10 µg/mL poly (I:C) was added for 24 h. (**D and E**) Total RNA was isolated for examination of *IFN*β expression by qPCR and compared to control cells. (**F–I**) Keratinocytes were infected for 6 h with *P. gingivalis* strain W83 or ∆K∆RAB and exposed to OMV and then stimulated with 50 µg/mL poly (I:C) for 24 h. Further (**F**) IFNλ, (**G**) IP-10, (**H**) RANTES were measured in culture media, whereas (**I**) *IFIT1* mRNA expression was determined in cell lysates using qPCR method. Results were presented as a mean of three independent experiments ± SD. OMV, outer membrane vesicle; *, *P* < 0.05; **, *P* < 0.01; ***, *P* < 0.001; ****, *P* < 0.0001; ns, non-significant.

The control of viral infection development depends on the proper functioning of the interferon response to non-self-nucleic acids (22). Therefore, we first determined whether *P. gingivalis* infection alters this response in epithelial cells lining the mucosal membrane, which serves as the entrance gate for viruses. For this purpose, we used oral epithelial cells and a synthetic analog of double-stranded RNA (dsRNA), which induces the activation of all pathways: TLR3, MDA5, and RIG-I, triggering the transcription of interferon pathway genes through IRF3 (Fig. 1A). It is worth emphasizing that IRF3 controls the cellular response to most clinically relevant viral infections in humans, including those related to the development of periodontitis (23). We established that dsRNA-dependent expression of IFNβ is inhibited shortly after bacterial infection (2 and 6 h), and the effect is maintained up to 24 h post-infection (p.i.) (Fig. 1B). Furthermore, the signal does not recover up to 24 h after exposition to poly (I:C) (Fig. 1B). This suggests that the observed effect is not temporary but rather long lasting.

Periodontal bacteria grow embedded in the structure of subgingival biofilm, but they can exhibit systemic effects secreting the outer membrane vesicles (OMVs) (24). Therefore, we examined the influence of OMVs on the studied phenomenon, demonstrating that their presence is sufficient to diminish interferon response of epithelial tissue (Fig. 1C, D, and F). Given that gingipains are the major executors of *P. gingivalis*-derived OMV activity, we used KYTs, highly selective inhibitors of those enzymes and showed that the proteolytic activity of OMVs determines the signal attenuation (Fig. 1D). This finding was confirmed in an infection model using a triple mutant ∆K∆RAB, devoid of all gingipains expression (Fig. 1C, E, and F). We also confirmed the biological significance of our observation examining the expression of interferon-dependent genes with antiviral activity IP-10, RANTES, and *IFIT1* (Fig. 1G through I). The above results show that the attenuation of the interferon response during *P. gingivalis* infection depends on the activity of gingipains.

### Kgp activity impairs the interferon response of epithelial cells

**Fig 2:**
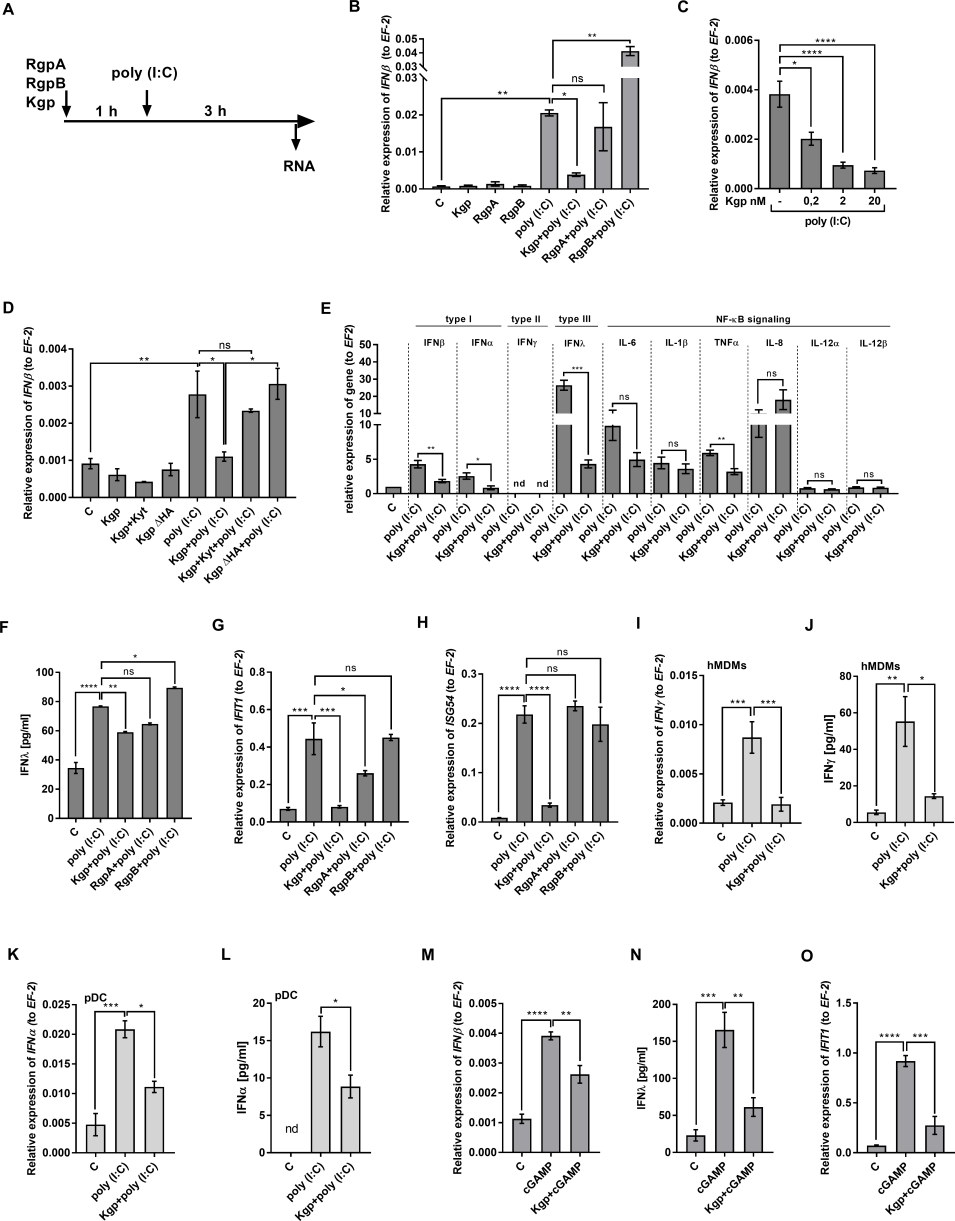
Kgp activity impairs the interferon response of gingival tissue. (**A**) Gingival keratinocytes were preincubated with gingipains for 1 h, then were exposed to 10 µg/mL poly (I:C), and total RNA was isolated upon 3 h. (**B**) Telomerase-immortalized gingival keratinocytes (TIGKs) were treated with 2 nM RgpA, RgpB, or Kgp before incubation with dsRNA or (**C**) pretreated with 0.2; 2, and 20 nM of Kgp. *IFN*β expression was determined using quantitative real-time PCR (qPCR) and was compared to (**B**) not stimulated cells or (**C**) poly (I:C)-treated cells. (**D**) The role of catalytic activity and hemagglutinin domain of Kgp in the expression of *IFN*β in dsRNA-stimulated cells was examined using 2 nM Kgp preincubated with KYT inhibitors and 2 nM (∆HA) Kgp without hemagglutinin domain. (**E**) The level of IRF and NF-kB-controlled cytokines mRNA was determined in keratinocytes, and relative expression in comparison to control equal to 1 was presented. (**F**) IFNλ protein level was estimated in culture media upon prestimulation with gingipains for 6 h and exposition of TIGKs to 50 µg/mL poly (I:C) for another 24 h. Next, cells were lysed, and (**G**) *IFIT1* and (**H**) *ISG54* gene expression was determined. Expression of *IFN*γ mRNA (**I**) in cell lysates and (**J**) IFNγ protein secretion in culture media from human monocyte-derived macrophages was determined in cells upon treatment with 2 nM Kgp for 1 h, what was followed by 3 h stimulation with 10 µg/mL poly (I:C). *IFNα* gene (**K**) and protein (**L**) expression in dendritic cells exposed to 2 nM Kgp and 10 µg/mL poly (I:C) was determined by qPCR and ELISA method, respectively. (**M**) The level of *IFN*β mRNA was examined in TIGKs preincubated for 1 h with Kgp and further exposed to 25 µg/mL cGAMP for 3 h. (**N**) IFNλ secretion and (**O**) *IFIT1* gene expression were examined upon 1 h incubation with 2 nM Kgp, followed by 24 h exposition to 25 µg/mL cGAMP. Results were presented as a mean of three independent experiments ± SD compared to control cells. *, *P* < 0.05; **. *P* < 0.01; ***, *P* < 0.001; ****, *P* < 0.0001; ns, non-significant.

To determine which gingipain is indispensable in the inhibition of dsRNA-dependent IFNβ production, we exposed epithelial cells to purified gingipains, including two arginine-specific proteases (RgpA and RgpB) and a lysine-specific protease (Kgp) (Fig. 2A). All enzymes were applied in non-toxic concentration for epithelial cells (range 0.2–20 nM) as we documented before (25). We observed significant reduction of interferon β expression but only in the presence of the lysine-specific protease—Kgp (Fig. 2B), as the arginine-specific proteases (RgpA, RgpB) did not affect IFNβ expression in poly (I:C)-stimulated cells. The effect of attenuating IFNβ expression by Kgp was dose dependent and observed even at a 0.2 nM concentration of enzyme (Fig. 2C).

Kgp is composed of catalytic and hemagglutinin domains (26), and both are considered as important for the biological role of this cysteine protease as a virulence factor (27, 28). Therefore, we used Kgp inhibited by the specific inhibitor KYT-36 and a recombinant form of the enzyme devoid of the hemagglutinin domain (∆HA Kgp). Inhibition of enzymatic activity by KYT and/or the absence of the hemagglutinin domain from Kgp reversed the attenuation of IFNβ expression in epithelial cells treated with poly (I:C) (Fig. 2D). Then, we investigated if reduced responsiveness of epithelial cells to poly (I:C) in the presence of Kgp is a general phenomenon or if it is restricted to the interferon pathway. We found that Kgp affects, specifically, interferon signaling, including all types of interferons, except IFNγ, which was below the detection limit (Fig. 2E). On the contrary, the majority of NF-κB-controlled transcripts, except *TNF*α*,* were not significantly changed (Fig. 2E). Since IFNλ expression is characteristic for epithelial cells (20, 29), we confirmed its regulation on protein level (Fig. 2F). The significance of the above phenomenon was reflected in the reduced production of *IFIT1* and *ISG54* mRNA, coding executive, antiviral protein, which directly affects virus recognition (30) (Fig. 2G and H). The interferon response of the mucous membranes is determined not only by the activity of epithelial cells but also by residual leukocytes (31, 32). Our study revealed that Kgp interferes with interferon pathways also in human macrophages (Fig. 2I and J; Fig. S1A), reducing expression of IFNγ and in dendritic cells reflected by downregulation of IFNα and their activation (Fig. 2K and L; Fig. S1B). Together, the above data indicate the broad biological impact of Kgp in the studied phenomenon. Confirmation of this thesis is the result documenting the reduction of the epithelial cell response to other agonists that mimic nucleic acids, including cGAMP in the presence of Kgp (Fig. 2M through O). Taken together, obtained data indicate that Kgp attenuates IRF signaling pathways, inhibiting interferon expression in response to nucleic acids. The process is universal for different cell types and might increase the susceptibility of periodontitis patients to viral infections. Collectively, we show that Kgp is a molecular tuner of interferon response during *P. gingivalis* infection.

### Kgp degrades key components of interferon response to dsRNA

**Fig 3:**
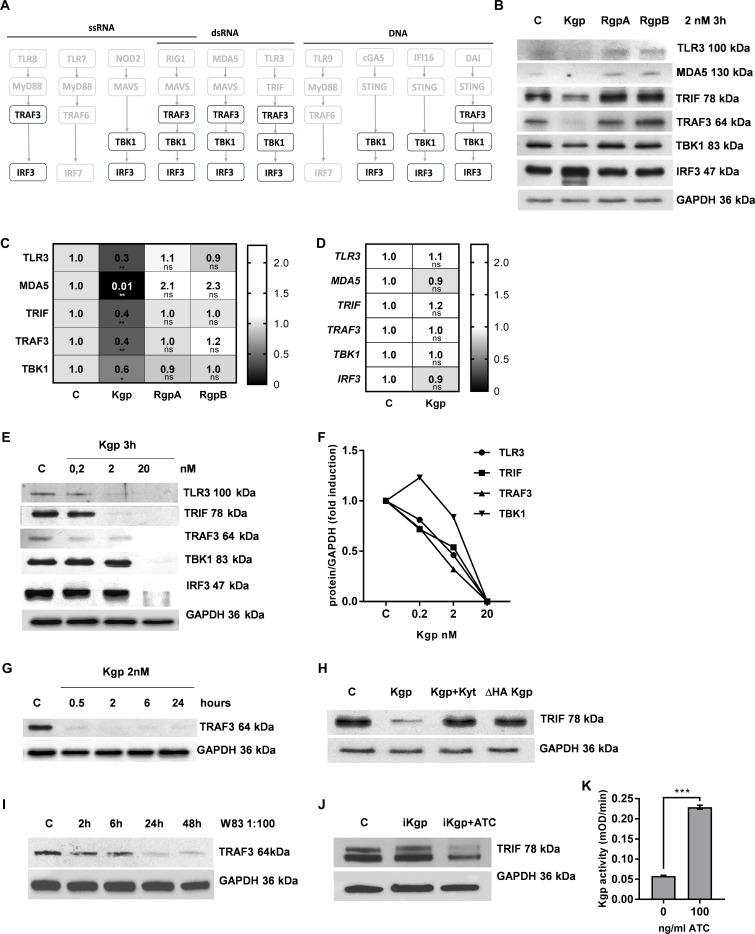
Kgp degrades key components of interferon response in gingival cells. (**A**) The scheme depicts common signaling pathway components involved in response to DNA and RNA recognition. (**B**) Gingiva cells were incubated for 3 h with 2 nM RgpA, RgpB, and Kgp. Western blot analysis of TLR3, MDA5, TRIF, TRAF3, TBK1, IRF3, and GAPDH proteins was conducted, and the representative result was presented. (**C**) The ratio of densitometry results of examined proteins expression to GAPDH from three independent experiments was presented as a heatmap. (**D**) TIGKs were incubated with 2 nM Kgp for 3 h, and total RNA was isolated. Expression of *TLR3, MDA5, TRIF, TRAF3, TBK1, IRF3,* and *EF2* genes was determined using quantitative real-time PCR. Results from three independent experiments were presented in the form of a heatmap. (**E**) Keratinocytes were exposed to 0.2, 2, and 20 nM Kgp for 3 h, and expression of proteins from TLR3 signaling pathway was determined. Representative result of Western blot analysis was shown. (**F**) The ratio of densitometry values from the presented Western blot analysis was depicted on the graph. (**G**) Expression of TRAF3 protein in the TIGKs stimulated with 2 nM Kgp for 0.5, 2, 6, and 24 h was presented. (**H**) The level of TRIF protein after incubation of cells with 2 nM Kgp, Kgp with KYT, and (∆HA) Kgp for 3 h was determined by Western blot analysis. A representative result is depicted. (**I**) Expression of TRAF3 protein in TIGK infected with *P. gingivalis* W83 strain for 2, 6, 24, and 48 h was examined, and representative results of Western blot analysis were shown. (**J**) TRIF protein expression was determined in cells that were infected with bacteria without Kgp expression (iKgp) and with Kgp induced by 100 ng/mL ATC. The protein level was compared to GAPDH expression. A representative result was presented. (**K**) Kgp activity was measured 24 h upon adding 100 ng/mL ATC to *P. gingivalis* DM105 strain growth media using Ac-Lys-pNa substrate (400 µM). Bars show mean values ± SD from three experiments.

To identify the molecular mechanism underpinning the Kgp-dependent attenuation of the interferon response, we examined the stability of common signaling components TRAF3, TBK1, and IRF3 (Fig. 3A bold text), and determined the cell response to major viral analogs: ssRNA, dsRNA, and DNA. The analysis was expanded on key molecules engaged in the signal transduction from the recognition of dsRNA by TLR3, MDA5, and TRIF. We found that the exposition of epithelial cells to Kgp resulted in proteolysis of all tested molecules (Fig. 3B and C). This phenomenon can be explained by the documented ability of Kgp to penetrate intracellularly without compromising the viability of epithelial cells (33). The effect was specific for Kgp, as arginine-specific gingipains (RgpA, RgpB) do not affect examined proteins (Fig. 3B and C). Moreover, we excluded the possibility that observed downregulation (Fig. 3B and C) is related to the decrease of gene expression exerted by Kgp activity (Fig. 3D). Additionally, degradation of TBK1 and IRF3 was also observed in macrophages, suggesting that the mechanism of Kgp intracellular protein degradation is universal (Fig. S2A). Next, we showed that Kgp-dependent degradation of intracellular proteins was dose dependent and observed already at 2 nM concentration of enzyme (Fig. 3E and F). The process was rapid and lasted for over 24 h (Fig. 3G). Moreover, the observed reduction in protein stability was dependent on both catalytic and hemagglutinin domains of Kgp (Fig. 3H). The relevance of the above observation was confirmed by degradation of TRAF3 in gingival keratinocytes exposed to *P. gingivalis*. The reduction of TRAF3 was noticed already 2 h post-infection and lasted up to 48 h (Fig. 3I). To show the importance of enzymatic activity of Kgp, we used a genetically modified strain of *P. gingivalis*, which produces Kgp upon exposition to anhydrotetracycline (ATC) (Fig. S2B and C). We observed that TRIF protein levels were downregulated only in ATC-treated cells (Fig. 3J), where Kgp was activated (Fig. 3K). To sum up, catalytically active Kgp specifically attenuates interferon signaling in gingival cells.

### Kgp upregulates replication and spread of HSV-1 virus in gingival keratinocytes

**Fig 4:**
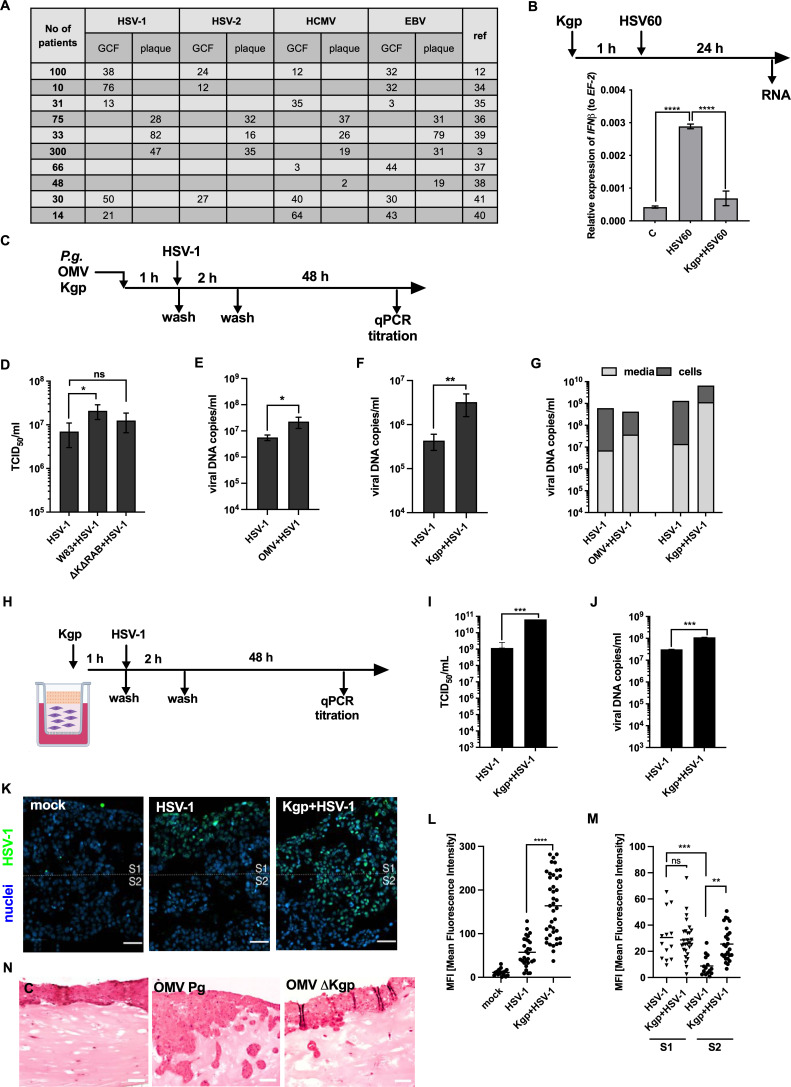
Kgp promotes HSV-1 replication. (**A**) Percent of herpesviruses positive probes from dental plaque and GCF obtained from patients suffering from periodontal disease was presented on the table prepared on the base of literature data. (**B**) Keratinocytes were exposed to 2 nM Kgp for 1 h and further stimulated with 5 µg/mL HSV60 DNA particles for 24 h. *IFN*β expression was estimated using quantitative real-time PCR (qPCR) and compared to not stimulated cells. (**C**) The timeline depicts experimental procedures (**D–G**). Cells were (**D**) infected with *P. gingivalis* W83 and ∆K∆RAB strain or stimulated with (**E**) OMV and (**F**) Kgp for 1 h. Further telomerase-immortalized gingival keratinocytes were infected with HSV-1 for 2 h, washed, and the results of (**D**) virus titration and (**E and F**) the number of viral DNA copies were determined upon 48 h. (**G**) The copies number of viral DNA particles inside the cells and released into the media in keratinocytes exposed to OMV and Kgp from representative experiment was presented. (**H**) The scheme depicts the experiment procedure. Kgp was incubated with cells in an OTG model for 1 h, which was followed by HSV-1 infection for 2 h and washing. After 48 h, (**I**) virus titration was conducted, and (**J**) the number of viral DNA copies was determined using qPCR. (**K**) Replication and penetration of the HSV-1 in the OTG model were visualized using antibodies against HSV-1 (green) and DAPI (nuclei, blue). The scale bar represents 50 µm. (**L**) Green fluorescence was quantified using ImageJ software and is presented as mean ± SD. (**M**) The signal intensity from HSV-1 was measured in two layers (S1 and S2) of OTG (zones separated with white lanes on L) and was presented on the graph. (**N**) Representative result of hematoxylin-eosin staining of OTG exposed to OMV isolated from *P. gingivalis* W83 strain and OMV from ∆Kgp *P. gingivalis*. The scale bar represents 50 µm. EBV, Epstein-Barr virus; HCMV, human cytomegalovirus; GCF, gingival crevicular fluid; *, *P* < 0.05; **, *P* < 0.01; ***, *P* < 0.001; ns, non-significant.

As we showed that *P. gingivalis* infection attenuates the interferon response of cells by Kgp-dependent degradation of crucial components of antiviral signaling pathways, thus, we intend to determine how this process translates to viral replication. For the study, we selected HSV-1, which is identified in plaque and gingival crevicular fluid (GCF) with other viruses from *Herpesviridae* family in periodontitis patients (Fig. 4A) (3, 12, 34–41). Moreover, it is worth noting that HSV-1 antibodies were identified in 36% of 5,266 periodontitis patients examined (42). Briefly, we demonstrated that the interferon response induced by the genetic material corresponding to HSV-1 is attenuated in epithelial cells exposed to Kgp (Fig. 4B). Then, we examined the influence of *P. gingivalis* infection on HSV-1, demonstrating that the W83 strain led to enhanced virus replication in the process dependent on gingipains expression (Fig. 4C and D). We confirmed this observation using OMVs and purified Kgp (Fig. 4C through F). Interestingly, we observed the total increase in the yield of virus with a significantly higher number of released virions into the medium in the response to cells treatment with OMV and Kgp (Fig. 4G). The enhanced course of viral infection was confirmed using an organotypic gingiva model (Fig. 4H) resembling the human tissue (43). We showed that the number of DNA copies and infectious viral particles increased in cultures exposed to Kgp (Fig. 4I and J). Moreover, visualization of the tissue model showed that Kgp not only increased the number of HSV-1-infected cells but also resulted in notable virus penetration into deeper tissue layers (Fig. 4K through M). The effect of the virus spread can also be explained by change of the tissue structure upon its exposition to OMV isolated from the *P. gingivalis* W83 strain in contrast to OMV isolated from the Kgp-null ∆*kgp* strain (Fig. 4N). Taken together, we showed that *P. gingivalis* infection facilitates the replication and spread of the virus in the gingival tissue, and the process is determined by Kgp secretion.

### Kgp causes the reactivation of latent form of the HSV-1 virus

**Fig 5:**
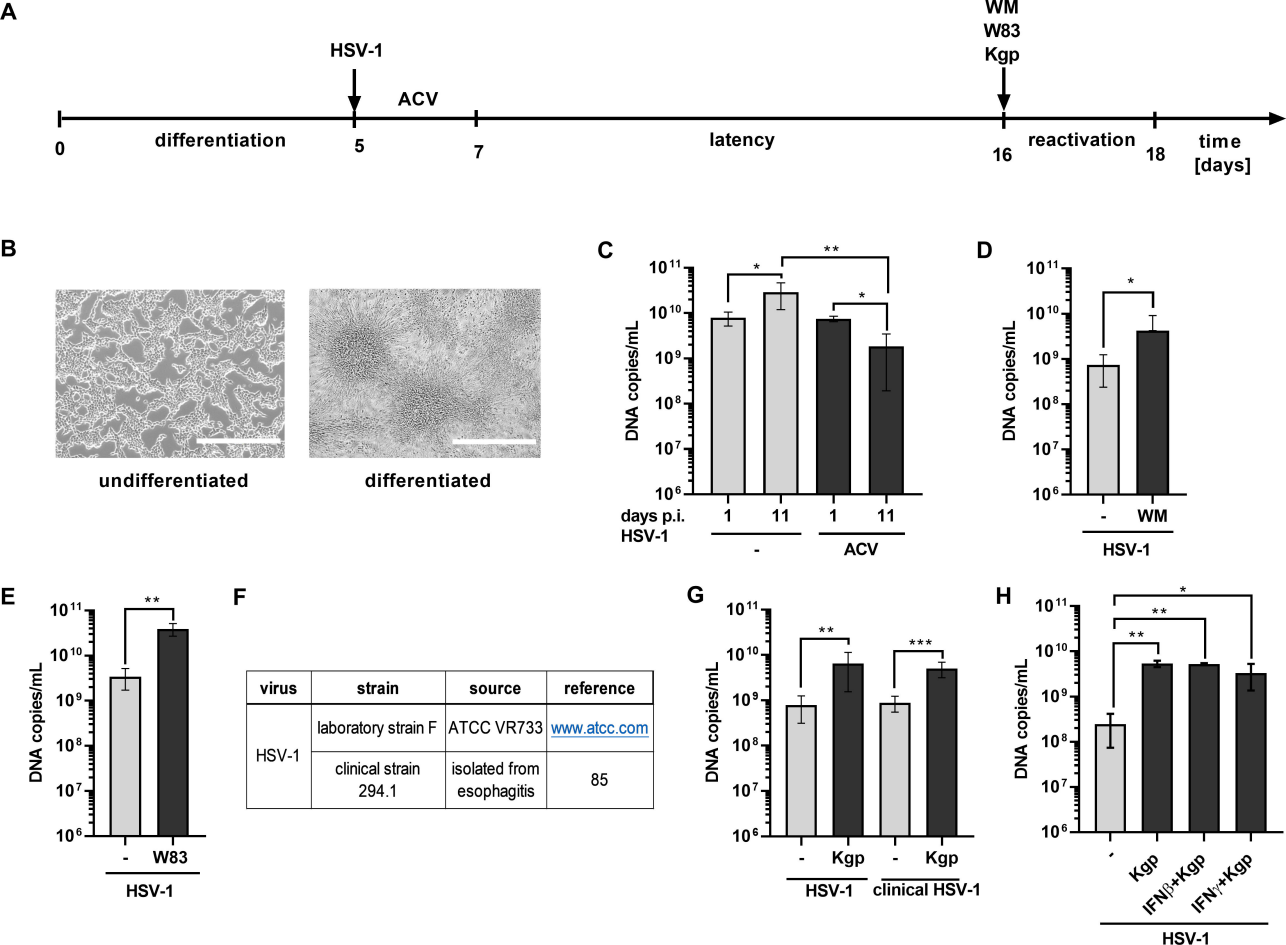
*P. gingivalis* reactivates latent form of the HSV-1. (**A**) The scheme depicts a timeline of LUHMES cells differentiation, HSV-1 latent phase induction, and reactivation. (**B**) Light microscopy pictures of LUHMES cells confirming their differentiation to post-mitotic neurons. (**C**) The number of viral DNA copies of HSV-1 ATCC strain was determined upon 1 and 11 days upon infection and exposition of cells to 50 µM acyclovir (ACV). Non-stimulated cells served as control. (**D**) Reactivation of the HSV-1 ATCC strain upon stimulation for 48 h with 1 µM wortmannin in neuronal cells was confirmed by the determination of viral DNA copies. (**E**) Neuronal cells with HSV-1 ATCC strain in latent phase were exposed to *P. gingivalis* cultivation media for 24 h, and viral DNA copies were estimated. (**F and G**) LUHMES cells infected with latent form of HSV-1 ATCC and clinical strain 294.1 were incubated with 20 nM Kgp for 24 h. (**H**) Neuronal cells infected with latent form of HSV-1 ATCC were incubated for 24 h with 20 nM Kgp or Kgp with 100 U/mL IFNβ or 100 U/mL IFNγ. (**G and H**) Viral DNA copies were measured in the LUHMES media. Results from two to three independent experiments were presented as mean ± SD. *, *P* < 0.05; **, *P* < 0.01; ***, *P* < 0.001; ns, non-significant.

According to World Health Organization (https://www.who.int/news/item/01-05-2020-massive-proportion-world-population-living-with-herpes-infection), HSV infection is common and affects approximately 67% of people under the age of 50, mostly remaining asymptomatic or unrecognized. Our data indicate that Kgp affects the lytic form of HSV-1, but the virus can persist typically in the body in a latent form within microglia cells. Given the proven presence of *P. gingivalis* bacteria and its virulence factors in the brain tissues of individuals suffering from Alzheimer’s disease (AD; [44]), we investigated whether Kgp could also reactivate HSV-1 in neuronal cells. In our experiments, differentiated Lund human mesencephalic (LUHMES) neuronal cells were infected with HSV-1, then induced latency using acyclovir (Fig. 5A and B). To verify the efficiency of latency, we compared the number of HSV-1 DNA copies on 1 and 11 days post-infection in the presence or absence of latency-inducing acyclovir (Fig. 5C). The process of virus reactivation was induced upon applying wortmannin, which selectively inhibits the PI3K pathway, a kinase crucial for HSV-1 persisting in latency (Fig. 5D) (45). The same phenomenon, virus reactivation, was observed once neuronal cells with the latent form of the virus were exposed for 24 h to *P. gingivalis* (Fig. 5E). As the potential for latency and viral reactivation strongly depends on viral strain (46), therefore, to confirm the role of Kgp, we applied the model of infection using ATCC HSV-1 and clinical 294.1 HSV-1 strain described as susceptible to latency (47) (Fig. 5F and G). We observed an increase in the number of DNA copies, revealing the reactivation of the virus in response to Kgp.

Reactivation of HSV-1 is known to strongly depend on the PI3K/Akt signaling pathway (45), while only limited evidence supports a role for interferons in this process (48). In our model, we also excluded the involvement of interferon signaling in Kgp-mediated HSV-1 reactivation, as Kgp treatment did not alter the expression of interferon-related genes (Fig. S3A and B). Moreover, supplementation of neuronal cells with IFNβ and/or IFNγ did not reverse the effects of the *P. gingivalis* enzyme (Fig. 5H). Additionally, Kgp is known to penetrate the cytosol of epithelial cells and promote degradation of Akt. Taken together, our data identify Kgp as a novel HSV-1 reactivation factor in ganglionic cells, which impairs cell signaling acting via an interferon-independent mechanism.

## DISCUSSION

Since the COVID-19 pandemic, more attention has been paid to viral infections and their long-term consequences, including the risk of developing concomitant diseases (49). Therefore, identifying the causes of increased susceptibility to viral diseases within the population has become an important topic. The risk of virus infection increases with the presence of chronic conditions, including asthma (50), diabetes (51), and rheumatoid diseases (52). However, a particularly significant role is played by disorders accompanied by microbial dysbiosis, such as colitis (53), bacterial vaginosis (54), and periodontitis (55), where the mucosa is significantly altered by pathogens inhabiting these niches. A growing body of clinical and epidemiological data indicates the particular influence of periodontal disease, which affects over 30% of the population in developed countries and which is classified as the sixth most prevalent disease worldwide, on the development of viral infections (56). Patients with periodontitis are generally more susceptible to viral infections of the upper and lower respiratory tract, like SARS-CoV-2, HIV-1, HBV/HCV, and IVA/B (7–9, 55). Considering this, increased prevention and/or management of periodontal disease is a crucial strategy for reducing the risk of viral disorders. However, the first step is to clarify the molecular basis of periodontal pathogens’ involvement in the development of the viral infection, the state of latency, and the spread of the viruses. We focused our study on the biology of the HSV-1 virus, as extensive clinical studies have shown that herpesviruses are the most commonly detected viruses in patients suffering from periodontal diseases (3, 10–12). Our results demonstrate, for the first time in the human gingival tissue, the propagation of the virus in the presence of a major periodontal pathogen, *P. gingivalis*. This provides evidence for the causal relationship suggested by clinical observations and explains the related epidemiological findings. Notably, another leading periodontal pathogen, *Aggregatibacter actinomycetemcomitans*, primarily associated with the acute form of periodontitis, has also been shown to enhance HSV-1 replication in oral epithelial cells, further confirming the link between periodontitis and *Herpesviridae* infections (57). Other scientific findings corroborate the role of periodontal pathogens, though they are not specifically related to *Herpesviridae*. For example, one study suggested that the exacerbation of COVID-19 infection is associated with periopathogens that increase the expression of ACE2 on epithelial cells, facilitating viral binding (58). Another study demonstrated that anaerobic bacteria colonizing the periodontium induce the expression of pro-inflammatory cytokines in the lower respiratory tract and promote the production of host proteases, thereby enhancing viral outcomes (59).

The enhanced propagation of the virus during coinfection can be attributed to the modulation of cellular signaling machinery by bacterial pathogens (60). Among the most critical pathways involved is the regulation of the interferon response. Notably, only type III IFN has been described as being modulated by periopathogens to such an extent that it alters viral propagation (20). Rodriguez-Hernandez et al. revealed an abolished IFNλ response to RNA and DNA analogs after cell exposition to *P. gingivalis (*2,0). Their findings demonstrated that this phenomenon is mediated by the reduced expression of transcription factors, including IRF1, IRF3, IRF7, and IRF9. This reduction can be partially explained by the proteolytic degradation of IRF receptors and subsequent inhibition of STAT1 phosphorylation. Furthermore, exposition to *P. gingivalis* blocks the nuclear translocation of the ISGF-3 complex, resulting in a diminished antiviral response. Our data confirmed clearly that attenuation of type III IFN is crucial for virus propagation, as demonstrated in epithelial cells. Limited information is available regarding the regulation of other interferon types by periopathogens. However, Mizraji et al. demonstrated that *P. gingivalis* ATCC 53977 disrupts IFN-I signaling by degrading MyD88 (18). The depletion of negative regulators of IFN-I signaling controlled by this adaptor molecule leads to prolonged IFNγ production. Our results obtained in a model of human macrophages and dendritic cells revealed that Kgp gingipain is among the bacterial factors that also significantly affect IFN type I and II. In this context, our findings significantly advance the understanding of the effects of *P. gingivalis* infection on IFN signaling, providing a comprehensive view for the first time. We demonstrate that key intracellular components—TRIF, TRAF3, TBK-1, and IRF3—essential for the type I interferon response are subject to proteolysis during infection with *P. gingivalis*. Our findings establish that the degradation of these proteins depends on the activity of the Kgp protease. Of note, in our experiments, we used physiologically relevant concentrations of gingipain (nM), which in the gingival crevicular fluids of periodontitis patients is estimated to be between ~100 nM and 1.5 µM (20, 61). This degradation attenuates cell signal transduction from all host receptors recognizing viral particles, including cGAS, IFI16, DAI, TLR3, MDA5, and RIG-1 (62). Besides, studies suggest that arginine gingipains, RgpA and RgpB, may also contribute to proteolysis of interferon pathway proteins, such as IFNAR and IFNLR (20). However, unlike Kgp, this process does not lead to attenuation of the antiviral response. The unique role of Kgp may be partly explained by the presence of its hemagglutinin domain, which enables efficient binding to the cell surface and subsequent penetration into the host cell cytosol, as documented in clinical studies by Booth and Lehner (63). Similarly, previous research by the Balkovetz group suggested that the hemagglutinin domain of Kgp facilitates the degradation of epithelial cell adherence junctions, potentially aiding *P. gingivalis* invasion into the periodontal connective tissue (28). Our findings align with this mechanism, as we showed that Kgp promotes the penetration of HSV-1 into deeper layers of the gingival tissue, using a human gingival tissue model. Additionally, enhanced distribution of Kgp via outer membrane vesicles amplifies its enzymatic activity, targeting cells of the mucosa-associated lymphoid tissue, including macrophages and dendritic cells (31). Considering that untreated periodontitis is a chronic disease, it can be hypothesized that prolonged exposition of gingival epithelium to *P. gingivalis* leads to long-term attenuation of the antiviral response, potentially with systemic implications. Our studies highlight the specificity and effectiveness of Kgp in regulating viral infections. It is as important as Kgp is constitutively expressed by the majority of *P. gingivalis* strains, and its enhanced production can be even promoted in the dysbiotic biofilm (64). In summary, we have uncovered a novel molecular mechanism of silencing the global antiviral response by Kgp-dependent degradation of key components of the interferon pathway.

The attenuation of interferon signaling may also benefit *P. gingivalis*, as the intracellular survival of this bacterium has been recently documented (65). The strategy of interferon suppression is a well-established virulence mechanism employed by other pathogens. For example, *Yersinia pestis* and *Shigella flexneri* modulate TLR4 activation and type I IFN induction (66, 67). *Legionella pneumophila* inhibits RLR activation (68), and *Yersinia pseudotuberculosis* disrupts MyD88- and TRIF-dependent signaling downstream of TLR4 (69). Therefore, the ability of *P. gingivalis* to modulate interferon signaling may represent a novel strategy to support its persistence within the intracellular niche.

Reactivation of the latent form of viruses is no less significant than the enhancement of their propagation. It has been established that the Epstein-Barr virus (EBV) can be reactivated by butyric acid produced by *P. gingivalis*, which increases histone acetylation and induces ZEBRA expression—a master regulator of the viral transition from latency to the lytic phase (70). Similarly, studies conducted by Chapple demonstrated that butyric acid from this periopathogen reactivates latent HIV in T-cells through histone modifications, contributing to AIDS progression (71). Additionally, the Kaposi’s sarcoma-associated herpesvirus (KSHV) is reactivated and disseminated via the p38 and JNK pathways in response to butyric acid and lipopolysaccharides derived from *P. gingivalis* (5). In this study, we reveal for the first time that in neuronal cells, which serve as reservoirs for latent HSV-1, the virus can be reactivated upon exposition to Kgp. Thus, Kgp not only facilitates the reproduction of the virus in the lytic phase of HSV-1 but also triggers reactivation from latency, further promoting viral dissemination. Reactivation of HSV-1 appears to depend on the inhibition of PI3K signaling (45). Constitutive activation of PI3K has been shown to contribute to severe KSHV and EBV infections (72). Since gingipains have been shown to negatively regulate PI3K/AKT activity (73, 74), this suggests that Kgp may lead to both the attenuation of viral infection and reactivation of the lytic form of the virus. However, as Dunn and Connor explain, the role of PI3K/Akt signaling in the antiviral response may vary depending on the stage of the viral life cycle and the specific virus strain (75). Interestingly, inhibition of PI3K signaling has been found to induce HSV-1 lytic gene expression in sensory and sympathetic neurons (76). We propose this pathway as an explanation for the observed reactivation of HSV-1 by Kgp in sensory ganglia cells rather than dependent on interferons. However, it remains unclear whether the effect of Kgp is direct or spontaneous, mediated through alterations in cellular signaling pathways, and this requires further investigation. Of note, recently published computational studies and clinical insights also suggest the role of Kgp in HSV-1 latency. These studies propose that *P. gingivalis* activates latent HSV-1 in periodontal tissues by inhibiting ICP4 expression and suppressing HSV-1 microRNA H6 (77). Furthermore, *in silico* data indicate the formation of a complex between Kgp and the ICP4 transcript, providing additional support for this mechanism.

Our discovery is particularly significant in light of evidence indicating the presence of *P. gingivalis* in the brains of patients suffering from periodontal disease (44). Notably, recent studies have identified HSV-1 as a putative causative agent of Alzheimer’s disease (78). Therefore, the *P. gingivalis*-dependent reactivation of HSV-1 might be considered a risk factor for the development of Alzheimer’s disease. Moreover, our findings provide a novel molecular explanation for the comorbidity between periodontal disease and AD. Furthermore, recent results have demonstrated the localization of gingipains in the substantia nigra of brains affected by Parkinson’s disease, and Kgp inhibitors have already entered human clinical trials for the treatment of dementia (79). Simultaneously, emerging data suggest that HSV-1 may play a role in the pathogenesis of Parkinson’s disease, potentially through modulation of the immune system (80, 81). Together, these findings imply that *P. gingivalis* infection could be a trigger for Parkinson’s disease development. However, this hypothesis requires further investigation.

**Fig 6:**
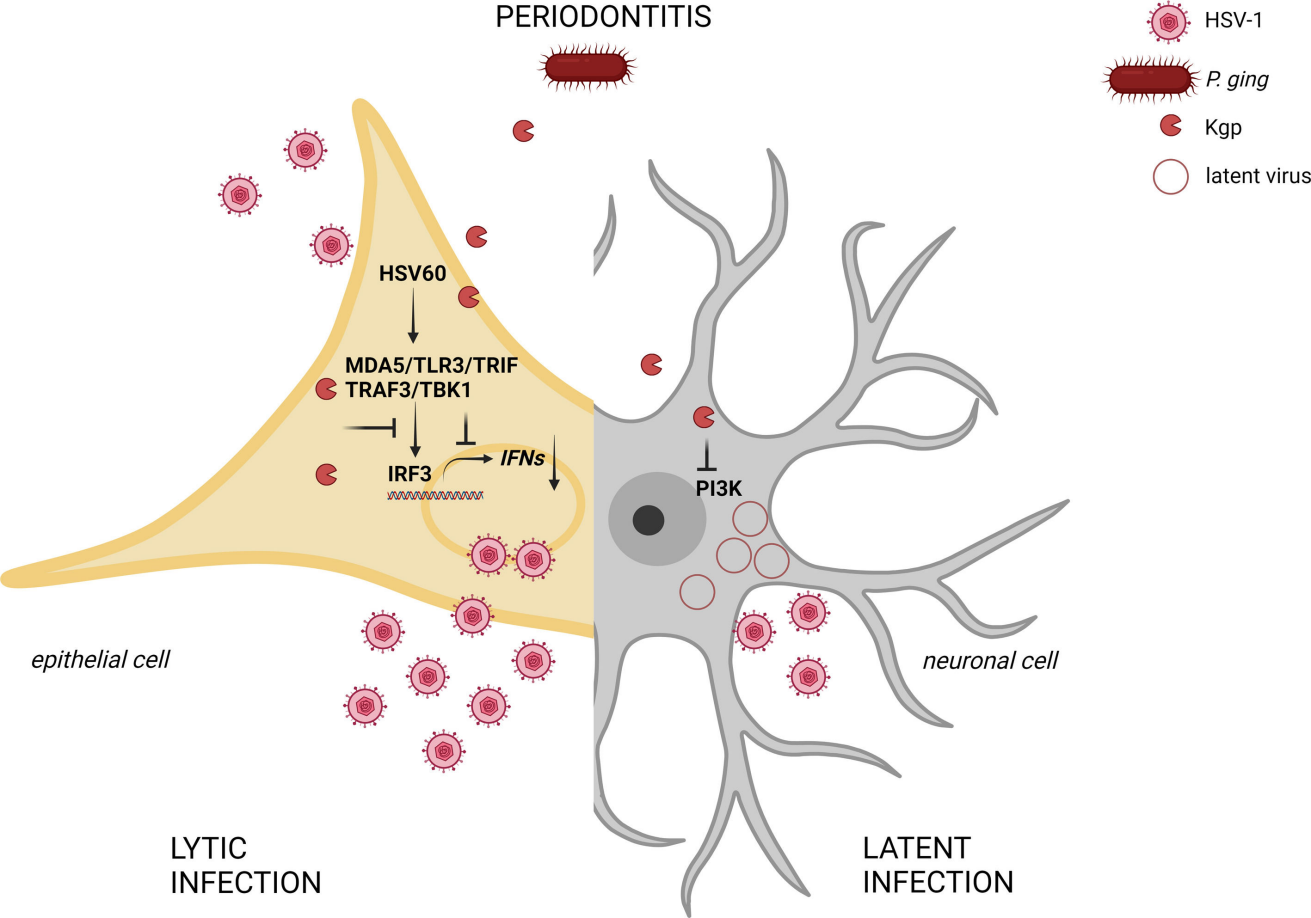
The role of gingipain Kgp from *P. gingivalis* in HSV-1 infection development. In mucosal epithelial cells of patients suffering from periodontitis, Kgp penetrates to the cytosol and catalyzes the cleavage of major components of interferon signaling, thus attenuating the antiviral response of the host. Attenuation of PI3K signaling (73) by Kgp in neuronal cells leads to the awakening of the latent form of the virus. Created in BioRender (J. Koziel, 2025, https://BioRender.com/e69x872).

In conclusion, our study demonstrates for the first time that *P. gingivalis* abolishes the interferon response in the epithelium of the oral mucosa. We identified lysine-specific protease Kgp as a key factor responsible for the proteolysis of molecules involved in antiviral signaling. Using a bacteria-virus co-infection model, we confirmed that periopathogens enhance HSV-1 replication and spread in the human gingival tissue and reactivate the latent form of the virus in neuronal cells (Fig. 6). These findings provide novel insights into the role of periodontal disease in the activation and exacerbation of viral infections. This underscores the critical importance of maintaining healthy gums to prevent viral diseases and their associated complications, while also highlighting new opportunities for the development of targeted therapies.

## MATERIALS AND METHODS

### Cells cultivation

Telomerase-immortalized gingival keratinocytes (TIGKs) were cultured at 37°C, 5% CO_2_ in KBM-Gold keratinocyte basal medium supplemented with Single Quots (Lonza) (82). Vero E6 cells obtained from ATCC collection (CRL-1586) were cultivated in Dulbecco modified Eagle medium (DMEM) supplemented with 3% heat-inactivated FBS, 100 U/mL penicillin, and 100 µg/mL streptomycin.

Human monocyte-derived macrophages were differentiated from peripheral blood mononuclear cells (PBMCs) isolated from human blood as described previously (83). Cells were seeded on 24-well plates in RPMI-1640 medium with 2 mM L-glutamine (Gibco) supplemented with 10% heat-inactivated autologous human plasma, 50 µg/mL gentamicin (Sigma), and incubated at 37°C, 5% CO_2_. Dendritic cells were isolated from PBMCs using BD IMAG Cell Separation System following a manufacturer’s protocol (BD Biosciences). Next, cells were seeded at 8 × 10^5^ per well in 200 µL of complete medium (RPMI 1640 medium supplemented with 10% FBS and 50 µg/mL gentamicin).

Blood was purchased from the Red Cross (Krakow, Poland) which de-identifies blood materials as appropriate for human subject confidentiality assurances. Thus, this manuscript adheres to appropriate exclusions from human subject approval.

### Bacterial culture

*P. gingivalis* wild-type strain W83 and mutant deprived of gingipains production **Δ**K**Δ**RAB were cultured at 37°C on 5% under anaerobic conditions (90% N_2_, 5% H_2_, 5% CO_2_) on sheep blood agar plates or in liquid trypticase soy broth (Sigma) supplemented with 50 µg/ml L-cysteine (Sigma), 5 µg/mL hemin (Sigma), and 0.5 µg/mL menadione (Sigma). The medium for ΔKΔRAB strain was additionally supplemented with 1 µg/mL tetracycline (Sigma). Overnight bacteria culture was centrifuged for 10 min at 5,000 RPM, and the bacterial pellet was washed three times and resuspended in PBS at a final optical density of 600 nm equal to 1, which corresponds to a density of 10^9^ CFU/mL.

### Preparation of *P. gingivalis* OMVs

Bacteria from an overnight culture were resuspended in PBS to an optical density (600 nm) equal to 1 and then were gently sonicated for 90 s to release OMVs to the cell supernatant. Further bacteria were removed by centrifugation at 10,000 RCF, 20 min, 4°C, and the remaining supernatant was subjected to ultracentrifugation at 150,000 RCF, 1 h, 4°C. The pellets containing OMVs were resuspended in the buffer containing 20 mM Bis-Tris, 150 mM NaCl, 5 mM CaCl_2_, at pH 6.8, and protein concentration was measured applying BCA protein assay (ThermoFisher). The activity of gingipains was determined using L-BApNA (for RgpA and RgpB) and N-(p-Tosyl)-Gly-Pro-Lys-4-nitroanilide (for Kgp) substrates.

### Infection of keratinocytes with *P. gingivalis*

Keratinocytes were seeded the day before experiments on 12-well plates in density of 0.8 × 10^6^ cells/well and were infected with *P. gingivalis* W83 or ΔKΔRAB at multiplicity of infection (MOI) 1:100 (cells to bacteria). Infected cells were then cultured for indicated time with poly (I:C) (10 or 50 µg/mL) and then lysed for RNA or protein isolation.

### Stimulation of cells with gingipains and pathogen recognition receptor agonists

Keratinocytes, human monocyte-derived macrophages, and dendritic cells were pre-stimulated for 1 h with activated gingipains at final concentrations of 0.2, 2, or 20 nM, and then cells were exposed to poly (I:C) (10 µg/mL, Sigma), cGAMP (25 µg/mL, Sigma), or HSV60 LyoVec (5 µg/mL, Invitrogen) for the indicated time. Culture media were collected, and cells were lysed for RNA and protein isolation. Activation of gingipains was proceeded for 15 min at 37°C in TNC buffer (100 mM Tris-HCl, 150 mM NaCl, 5 mM CaCl_2_, and 20 mM cysteine, pH 7.5) with 20 mM L-cysteine. In indicated experiments, gingipains and OMV were pre-incubated for 15 min at 37°C with specific inhibitors KYT-1 and KYT-36 in a final concentration of 1 µM. The lysin-specific gingipain without hemagglutinin domains was prepared as described here (84).

### Co-infection of keratinocytes with bacteria and virus

TIGKs were exposed for 1 h to *P. gingivalis* W83 and ΔKΔRAB strain, and then cells were infected with HSV-1 virus (TCID_50_ 400) or mock for another 2 h at 37°C, 5% CO_2_. Further keratinocytes were washed three times with PBS, and fresh media were added. Analysis of HSV-1 virus replication was carried out 48 h post-virus infection. After this time, media were collected, and cells were lysed using TRI reagent for further RNA isolation.

### Virus preparation, titration, and DNA quantification

HSV-1 virus strain F was obtained from ATCC collection VR733. Clinical HSV-1 strain 294.1 was isolated from esophagitis and kindly provided by Donald M. Coen from Department of Biological Chemistry and Molecular Pharmacology, Harvard Medical School, Boston (85). Viral stocks were prepared by infection of Vero E6 cells, which were lysed 48 h p.i. by two freeze-thaw cycles. The medium containing the virus was aliquoted and stored at −80°C. Non-infected cells were treated in the same manner to obtain mock. The yield of the stock and sample probes was evaluated by virus titration in 96-well plates on fully confluent Vero E6 cells, according to Reed and Muench method (86). The plates were incubated with titrated probes for 48 h at 37°C, and the cytopathic effect was scored using a microscope, and then TCID_50_ (50% tissue culture infection dose) was calculated.

Briefly, viral DNA was isolated from media and cell lysates using the Viral DNA/RNA Isolation Kit (A&A Biotechnology). The virus yield was verified by quantitative real-time PCR (qPCR) reaction proceeded in 10 µL mixture consisting of TaqManTM Fast Advanced Master Mix, TaqMan Microbe Detection assay against HSV-1 (Vi04230116_s1) and 1 µL of viral DNA. The temperature profile in CFX96 Touch Real-Time PCR Detection System (Bio-Rad) was 20 s at 95°C, followed by 40 cycles of 3 s at 95°C and 20 s at 60°C. HSV-1 DNA quantification standards were prepared as described here (87).

### ELISA assay

The levels of the cytokines IP-10 and RANTES in culture media were assessed using the CBA Human Chemokine Kit (BD Biosciences), following the manufacturer’s recommendations. Data acquisition was performed with the BD LSR Fortessa system (Becton Dickinson) and analyzed using BD FACSDiva software. The concentrations of human IFNλ (Biotechne) and IFNα (Proteintech) in the culture media were determined using commercially available enzyme-linked immunosorbent assays, in accordance with the manufacturers’ instructions.

### Quantitative reverse-transcription PCR

Total RNA was isolated from cells using TRI Reagent (Sigma) and reverse transcribed using the High Capacity cDNA Reverse Transcription Kit (Applied Biosystems) according to the manufacturer’s instructions. Five hundred nanograms of RNA was used for complementary DNA synthesis in a total volume of 20 µL. The quantitative PCR was performed in 15 µL, which included 1 µL of cDNA sample, 10 µM of forward and reverse primers, and 1 × GoTaq qPCR Master Mix (Promega). The primers and reaction conditions used in qPCR are listed in Table 1. Housekeeping gene *EF-2* was used for normalization. The reaction was initiated by 10 min denaturation and then was carried out for 40 cycles with a final elongation step at 72°C for 10 min. The cycle threshold (C_t_) values were determined and calculated using the ΔΔCt method. Melt curve analysis was performed to confirm the performed reaction’s specificity.

**TABLE 1:** List of primers for qPCR

Oligonucleotide	Sequence	Program
*IRF3 F* *IRF3 R* *IFN*γ *F* *IFN*γ *R*	5′ -TACGTGAGGCATGTGCTGA-3′ 5′ -AGTGGGTGGCTGTTGGAAAT-3′ 5′ -GGCTTTTCAGCTCTGCATCG-3′ 5′ -TTCTGTCACTCTCCTCTTTCCAA-3′	95°C, 30 s 58°C, 30 s 72°C, 30 s
*IL-8* F *IL-8* R *TRIF F* *TRIF R* *TRAF3 F* *TRAF3 R* *TBK1 F* *TBK1 R* *MDA5 F* *MDA5 R* *IFIT1 F* *IFIT1 R* *EF2* F *EF2* R	5′-ATGACTTCCAAGCTGGCCGTGGCT-3′ 5′-TCTCAGCCCTCTTCAAAAACTTCT-3′ 5′ -CCAAGCCATGATGAGCAACC-3′ 5′ -AGATCTGGGAGTGTTCGTCC-3′ 5′ -CTCACAAGTGCAGCGTCCAG-3′ 5′ -GCTCCACTCCTTCAGCAGGTT-3′ 5′ -CCCTTTGAAGGGCCTCGTAG-3′ 5′ -ACCCCGAGAAAGACTGCAAG-3′ 5′ -GTTGAAAAGGCTGGCTGAAAAC-3′ 5′ -TCGATAACTCCTGAACCACTG-3′ 5′ - AGCAACCATGAGTACAAATGG-3′ 5′ - TGTTTCACATAGGCTAGTAGG-3′ 5′-GACATCACCAAGGGTGTGCAG-3′ 5′ -TTCAGCACACTGGCATAGAGGC-3′	95°C, 30 s 60°C, 30 s 72°C, 45 s
*IL-6 F* *IL-6 R* *IL-1*β *R* *IL-1*β *F* *TNF-*α *F* *TNF-*α *R* *IL-12*α *F* *IL-12*α *R* *IL-12*β *F* *IL-12*β *R* *IFN*β *F* *IFN*β *R* *IFN*λ *F* *IFN*λ *R*	5′ -AAATTCGGTACATCCTCGACGGCA-3′ 5′ -AGTGCCTCTTTGCTGCTTTCACAC-3′ 5′ -GATGTCTGGTCCATATGAACTG-3′ 5′ -TTGGGATCTACACTCTCCAGC-3′ 5′ -GTCAGATCATCTTCTCGAACCCCGA-3′ 5′ -CAGGGCAATGATCCCAAAGTAGA-3′ 5′ -ATGATGGCCCTGTGCCTTAG-3′ 5′ -TCCGGTTCTTCAAGGGAGGA-3′ 5′ -ATTCTGCGTTCAGGTCCAGG-3′ 5′ -AGAACCTAACTGCAGGGCAC-3′ 5′ -CTTGGATTCCTACAAAGAAGCAGC-3′ 5′ -TCCTCCTTCTGGAACTGCTGCA-3′ 5′ -GAAGCAGTTGCGATTTAGCC-3′ 5′ -GAAGCTCGCTAGCTCCTGTG-3	95°C, 30 s 56°C, 30 s 72°C, 45 s
*IFN*α *F* *IFN*α *R*	5′ -GATACTCCTGGCACAAATGG-3′ 5′ -AAGGTCTGCTGGATCATCTC-3′	95°C, 30 s 55°C, 30 s 72°C, 45 s
*ISG54 F* *ISG54 F*	5′ -GGAGCAGATTCTGAGGCTTTGC-3′ 5′ -GGATGAGGCTTCCAGACTCCAA-3′	95°C, 30 s 62°C, 30 s 72°C, 45 s

### Western blot analysis

Keratinocytes were lysed in 120 µL of RIPA buffer containing 0.25% deoxycholate, 0.05% SDS, 0.5% Nonidet P-40, 2.5 mM EDTA, and freshly added protease inhibitors cocktail. The protein concentration was determined using the BCA protein assay, and equal amounts of the protein were subjected to 10% SDS-PAGE under reducing conditions. Further proteins were transferred to PVDF (Amersham) membrane in transfer buffer (25 mM Tris, 0.2M glycine, 20% methanol), and then membranes were blocked for 2 h at room temperature with 5% skim milk in TBST buffer (20 mM Tris, 0.5 M NaCl, 0.05% Tween 20, pH 7.5). Next, PVDF was incubated overnight with primary antibodies: rabbit anti-TRIF 1:1,000 (Invitrogen), rabbit anti-TRAF3 1:1,000 (Invitrogen), rabbit anti-TBK-1 1:1,000 (abcam), rabbit anti-IRF3 1:1,000 (abcam), rabbit anti-TLR3 1:500 (abcam), rabbit MDA-5 1:500 (Cell signaling), or mouse anti-GAPDH 1:10,000 (Cell signaling). Antigen-antibody binding was detected using HRP-conjugated secondary antibodies for 2 h at room temperature: goat anti-rabbit IgG (1:10,000, Cell Signaling). The signal was developed using Luminata Crescendo Substrate (Merck Milipore) and exposed to Medical X-ray film (Agfa).

### Induced Kgp expression

The system for induced gene expression in *P. gingivalis* was based on previously published *Bacteroides thetaiotaomicron* one (88). Briefly, a sequence of a promoter induced by anhydrotetracycline (promATc) and a repressor coding gene (TetR) were artificially synthesized and introduced by a homologous recombination before the Kgp coding sequence. For this, a previously constructed vector pNKgp-cep (89) was utilized. In the pNKgp-cep, the native Kgp promoter sequence was substituted by the promATc-TetR genetic element with the Gibson cloning method. The resulting plasmid pDM105 was introduced to electrocompetent *P. gingivalis* cells, and after selection on plates supplemented with ampicillin 2 mg/mL, the DM105 strain was obtained. All genetic manipulations were confirmed by sequencing.

### Kgp activity assay

To measure Kgp activity, *P. gingivalis* cells were inoculated from fresh plates into 3 mL of eTSB preculture and grown under anaerobic conditions. The next day, the cultures were diluted to OD_600_ = 0.1, and, where necessary, anhydrotetracycline was added to final concentrations of 100 ng/mL. After 24 h, samples were collected for analysis. In a 96-well format, 20 µL of the samples was preincubated in an assay buffer (200 mM Tris-HCl, 100 mM NaCl, 5 mM CaCl_2_, pH 7.6) supplemented with fresh L-cysteine (10 mM) before the addition of the Ac-Lys-pNa substrate (400 µM). The reaction was performed in a total volume of 200 µL. Changes in absorbance were monitored at 405 nm for 30 min at 37°C. The SoftMax Pro software was used to determine Kgp enzymatic velocity (mOD/min). The obtained data were normalized to 1 µL of a sample with OD_600_ = 1. The experiment was performed with two biological replicates and three technical replicates.

### Preparation of 3D gingiva model

The protocol of 3D culture preparation and media components was described previously (43). Briefly, 1.5 × 10^5^ gingival fibroblasts suspended in 10% FBS in DMEM were mixed with ice-cold Matrigel (Corning) in a final concentration of 7 mg/mL. The mixture was placed in the middle of 12-well cell culture inserts (Greiner) and incubated for 2 h at 37°C, 5% CO_2_ without culture medium, to allow the matrix to solidify. To the top of the inserts, 0.5 and 1.5 mL to the bottom DMEM culture medium supplemented with 10% FBS, 100 U/mL penicillin, and 100 ug/mL streptomycin were added to the inserts. After 24 h, the matrix circumference was outlined using a mini scalpel blade, and the medium was changed to the fresh one. Three days later, 1 × 10^6^ TIGK cells suspended in EPM1 were added to the top of the insert. On the ninth day after the start of the procedure, EPM1 was removed, and EPM2 was added to the bottom of the insert. The epithelial layer on the top was exposed to the air for epithelial differentiation and stratification. Ten days later, 3D cultures of gingiva were used for further experiments.

### Stimulation and HSV-1 infection of 3D model

On the top of the insert, PBS containing 20 nM of Kgp was applied, and tissue was incubated for 2 h at 37°C, 5% CO_2_, and cells were exposed to HSV-1 virus TCID_50_ equal to 400. Upon 2 h of infection, inserts were washed three times with PBS to remove the unbound virus, and the cultures were maintained at an air-liquid interface for the rest of the experiment. To determine HSV-1 replication 48 h post-infection, 200 uL of PBS was administrated to the apical surface of keratinocytes. After 15 min of incubation at 37°C, washes were collected for viral DNA isolation, and tissue was lysed using lysis buffer for further DNA isolation or preserved for immunofluorescent staining.

### Staining of 3D model

Cell inserts were fixed in 10% formaldehyde at 4°C for 1 h, and then, they were excised with a scalpel, washed with PBS, dehydrated with a graded series of ethanol (from 50% to 100%), and xylene. After embedding in paraffin, 3D cultures were cut into 5 µm sections and mounted on poly-L-lysine-coated glass slides and stained with hematoxylin and eosin solution. For immunohistochemical analysis, sections were dewaxed in xylene and rehydrated with a series of gradually increasing ethanol (from 100% to 50%). Slides were heated for 20 min in sodium citrate buffer (10 mM sodium citrate and 0.05% Tween 20 [pH 6.0]), incubated for 1 h at room temperature in blocking buffer (5% normal goat serum, 0.1% saponin in PBS), and stained first with antibodies against HSV-1 (Abcam) (1:100, 5% normal goat serum, 0.1% saponin in PBS) and after several washes with secondary antibodies goat anti-rabbit conjugated with Alexa Fluor 488 (1:500; Cell Signaling) for 45 min at room temperature. Finally, cell nuclei were stained with DAPI using ProLong Antifade Mounting Medium and were visualized with ZEISS Axio Observer 7 fluorescent microscope using ZEN software.

### LUHMES cells cultivation

Lund human mesencephalic (LUHMES) cells are human embryonic neuronal precursor cells, were purchased from Bio Cat collection (T0284-GVO-ABM) and cultured according to the supplier’s recommendations. Cell culture flasks and plates were coated with a mixture of poly-L-ornithine hydrobromide (Sigma) with fibronectin (Sigma) for 3 h at 37°C, rinsed with PBS, and allowed to fully air dry at room temperature before cell plating. Cells were grown in proliferation medium containing Dulbecco modified Eagle medium-F12, (ATCC) supplemented with 1% N_2_ (ThermoFisher Scientific), 1× penicillin-streptomycin-glutamine solution (ThermoFisher Scientific), and recombinant human fibroblast growth factor (Peprotech; 40 ng/mL final concentration) on 48-well cell plates (ThermoFisher Scientific). For cell differentiation, the medium was changed to DMEM-F12 supplemented with N_2_, tetracycline hydrochloride at a 1 ug/mL final concentration (Sigma), *N*6,2′-*O*-dibutyryladenosine 3′,5′-cyclic monophosphate sodium salt at a 1 mM final concentration (Sigma), and recombinant human glial cell-derived neurotrophic factor at 2 ng/mL final concentration (R&D Systems). Experiments were conducted 5 days upon induction of differentiation.

### Establishing latency and reactivation of HSV-1 in LUHMES cells

Differentiated neurons were treated with 50 µM acyclovir for 2 h and further infected with HSV-1 in a complete medium at an MOI of 3 corresponding to TCID50. Additionally, ACV was added to the virus inoculum. Two days later, the medium was changed without the addition of ACV, and neurons were cultured for 11 days p.i. with medium changes every 2 days. Reactivation was induced at day 11 p.i. by incubating infected neurons for 48 h with 1 µM wortmannin (Sigma), 20 nM Kgp, Kgp with IFNβ (Prospec) or IFNγ (Proteintech), and culture medium of *P. gingivalis* for 24 h. Media were collected, and cells were lysed for the establishment of HSV-1 DNA level at 1 day p.i., 11 days p.i., and upon virus reactivation.

### Statistical analysis

Statistical significance was calculated using Student *t*-test and one-way ANOVA. Data were presented as mean ± SD and considered as statistically significant for *P*-value of <0.05. GraphPad Prism 8.0 software was used for data analysis. Densitometry and fluorescence measurement were conducted using ImageJ software, and graphics were prepared applying Servier Medical Art and Biorender. The statistical significance was indicated as follows: * for *P* < 0.05; ** for *P* of < 0.01; *** for *P* < 0.001; ns for non-significant.

## Data Availability

All relevant data are within the article and its supplemental files.

## SUPPLEMENTAL MATERIAL

Figure S1 mbio.00298-25-s0001.tif Supplemental information for Fig. 2.

Figure S2 mbio.00298-25-s0002.tif Supplemental information for Fig. 3.

Figure S3 mbio.00298-25-s0003.tif Supplemental information for Fig. 5.

ASM does not own the copyrights to Supplemental Material that may be linked to, or accessed through, an article. The authors have granted ASM a non-exclusive, world-wide license to publish the Supplemental Material files. Please contact the corresponding author directly for reuse.
