# Supplementary figures and images for: Lys-specific gingipain (Kgp) of P. gingivalis promotes viral infection by disabling the interferon pathway
Source: mBio. 2025 Aug 28;16(10):e00298-25. doi: 10.1128/mbio.00298-25 (PMC12506115; doi:10.1128/mbio.00298-25)

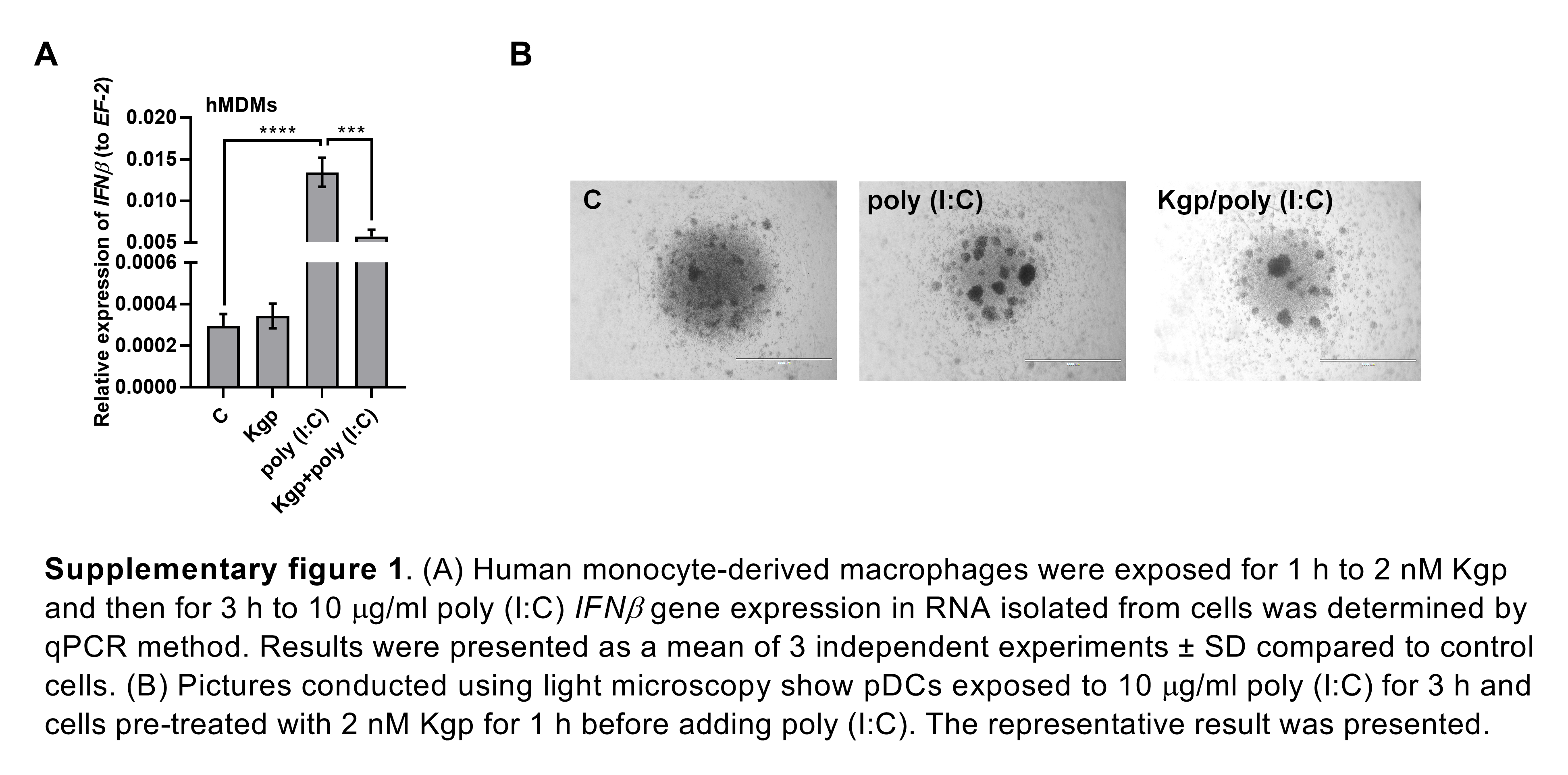

Supplement: Figure S1 — Supplemental information for Fig. 2. [file mbio.00298-25-s0001.tif]

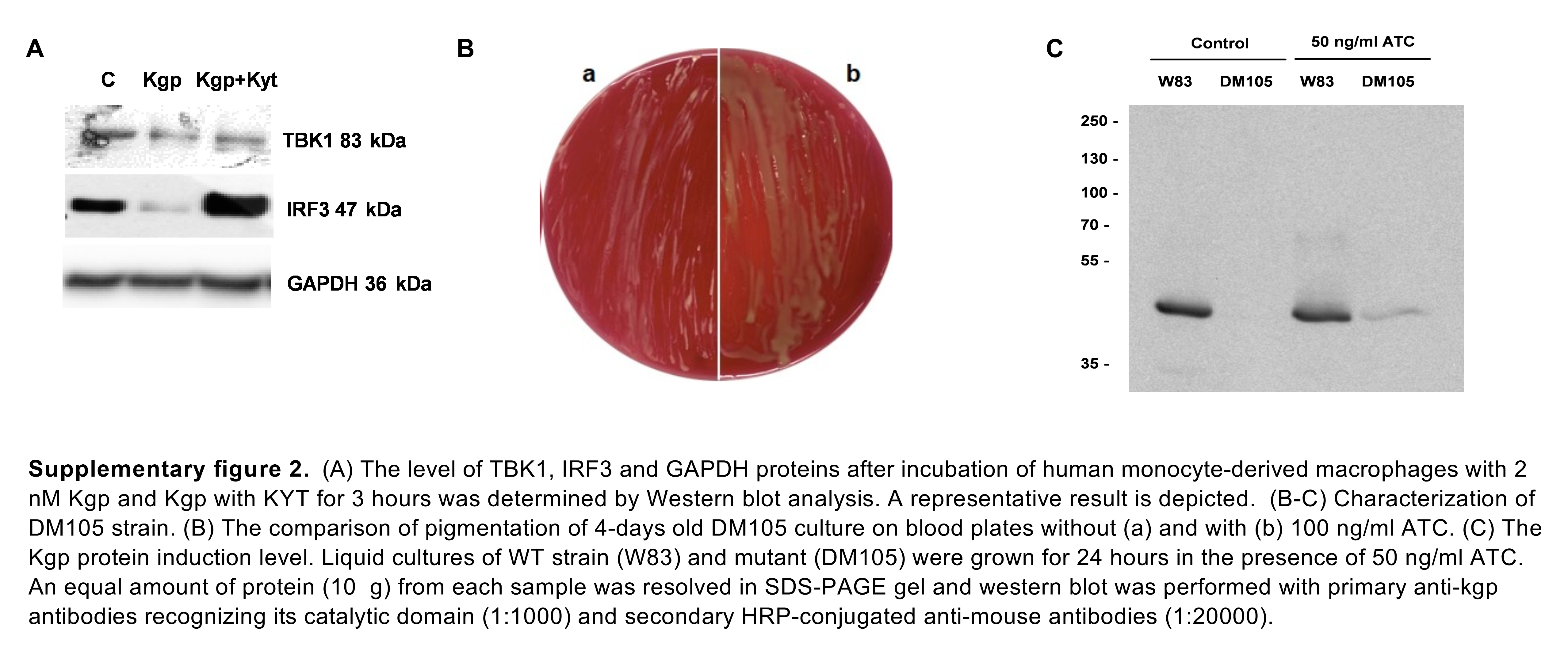

Supplement: Figure S2 — Supplemental information for Fig. 3. [file mbio.00298-25-s0002.tif]

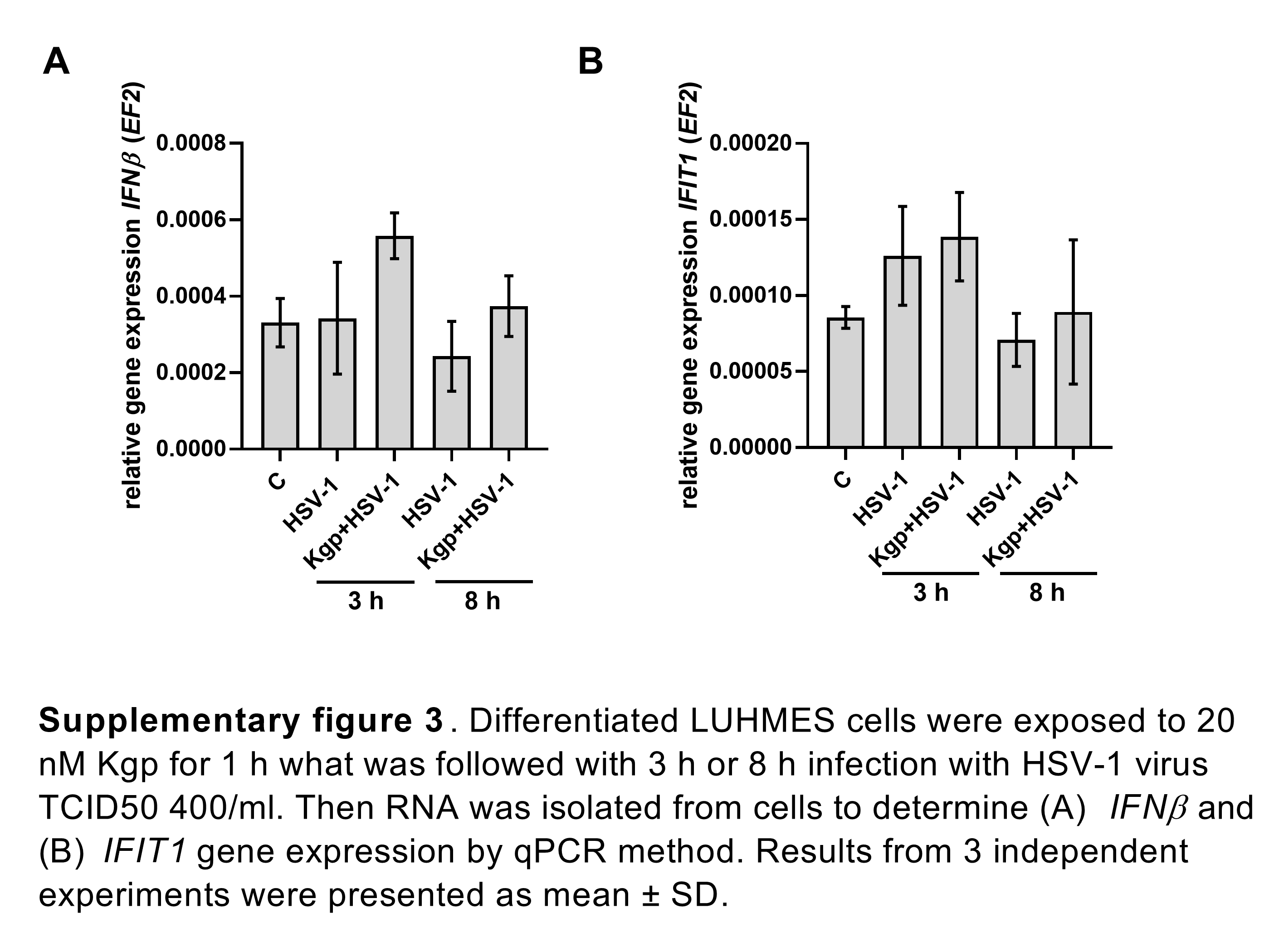

Supplement: Figure S3 — Supplemental information for Fig. 5. [file mbio.00298-25-s0003.tif]
